# Data-driven research and healthcare: public trust, data governance and the NHS

**DOI:** 10.1186/s12910-023-00922-z

**Published:** 2023-07-14

**Authors:** Angeliki Kerasidou, Charalampia (Xaroula) Kerasidou

**Affiliations:** 1 Ethox Centre, Oxford Population Health (Nuffield Department of Population Health, Big Data Institute, Li Ka Shing Centre for Health Information and Discovery Oxford, University of Oxford, Oxford, UK; 2 Ethox Centre, Oxford Population Health (Nuffield Department of Population Health, Big Data Institute, Li Ka Shing Centre for Health Information and Discovery Oxford, University of Oxford, Oxford, UK

**Keywords:** Trust, Data sharing, Research, Healthcare, Data governance

## Abstract

It is widely acknowledged that trust plays an important role for the acceptability of data sharing practices in research and healthcare, and for the adoption of new health technologies such as AI. Yet there is reported distrust in this domain. Although in the UK, the NHS is one of the most trusted public institutions, public trust does not appear to accompany its data sharing practices for research and innovation, specifically with the private sector, that have been introduced in recent years. In this paper, we examine the question of, what is it about sharing NHS data for research and innovation with for-profit companies that challenges public trust? To address this question, we draw from political theory to provide an account of public trust that helps better understand the relationship between the public and the NHS within a democratic context, as well as, the kind of obligations and expectations that govern this relationship. Then we examine whether the way in which the NHS is managing patient data and its collaboration with the private sector fit under this trust-based relationship. We argue that the datafication of healthcare and the broader ‘health and wealth’ agenda adopted by consecutive UK governments represent a major shift in the institutional character of the NHS, which brings into question the meaning of public good the NHS is expected to provide, challenging public trust. We conclude by suggesting that to address the problem of public trust, a theoretical and empirical examination of the benefits but also the costs associated with this shift needs to take place, as well as an open conversation at public level to determine what values should be promoted by a public institution like the NHS.

## Background

There seems to be wide acknowledgement of the importance of trust in the context of data driven research and healthcare. It has been argued that building trust in this area can facilitate public acceptability of data sharing and the adoption of new technologies such as AI [1, 2]. And yet, what it means, and how to promote trust in this context remains vague [3, 4]. In the UK, the NHS is one of the most trusted public institutions, and also the single biggest holder of health data worldwide [5]. This could create favourable conditions for the establishment and promotion of a robust data driven health research and innovation sector and of data driven healthcare, something that successive governments in recent years have been trying to achieve [6–8]. However, in recent years, data sharing initiatives as developed by successive governments and implemented through the NHS have been met with public distrust [9, 10]. One of the concerns cited is the involvement of private companies in the healthcare space, and worries of a potential “sell-off” of NHS patient data to for-profit companies for research and product development [11–14]. What it is about the sharing of NHS data with private for-profit companies in research and product development that is problematic and might be negatively affecting public trust is less clear. To answer this question, we first provide an account of public trust to help elucidate the kind of relationship it signifies between the public and health institutions, such as the NHS, and the kind of obligations and expectations that can be reasonably extrapolated from this relationship. Then we examine whether the way in which the NHS is managing patient data, and the particular collaborations and data sharing practices developed with the private sector fit under this trust-based relationship between the public and the NHS. We argue that the datafication of healthcare and the broader ‘health and wealth’ agenda adopted by consecutive UK governments represent a major shift in the institutional character of the NHS. This shift brings into question the meaning of public good that the NHS is supposed to provide, and thus challenges public trust. We suggest that in order to address the problem of public trust, a substantive examination – theoretical and empirical – of the benefits but also, and importantly, the costs associated with this shift needs to take place, as well as an open public conversation to determine what values should be endorsed and promoted by a public institution like the NHS.

## Public trust

A number of scholars have been discussing the issue of trust and data governance particularly in the context of healthcare [3, 4, 15, 16]. These discussions have engaged with the philosophical and sociological literature to understand and explain the role and importance of trust in this context, and draw meaningful distinctions between related concepts such as trust, trustworthiness, reliance, reliability and confidence [3, 15, 16]. A proper analysis of these terms fall outside the scope of this paper, yet a short clarification of these concepts might be of use here. Trust denotes a relationship where one has a reasonable belief in the other person’s ability and good will or expressed commitment to perform a certain action [17–20]. Reliance, and confidence – the latter, a term most often found in the sociological literature [21, 22] - confer the same reasonable expectation that an action will be performed, but do not entail affective elements, in the sense that there is no expectation of good will or expressed commitment [17, 20, 23]. Trustworthiness and reliability, on the other hand, refer to characteristics of the agent that is (could be) trusted or relied upon [17, 20, 24, 25]. As O’Neil notes, when it comes to trust, the crucial point is to be able to discern who is trustworthy and who is untrustworthy in order to be sure to place our trust appropriately [19, 25]. However, whilst using accounts of interpersonal trust to make sense of trust between collectives is useful [26], they do not capture the distinct and morally relevant context that frames the relationship between the public and public institutions, namely, the democratic structure that underpins this trust relationship. It is this political context, we maintain, that creates specific conditions both for the public and public institutions. When technological, policy or economic developments impact on the ways in which these rights and obligations are understood and practiced, the question arises of whether a rethinking of the relationship between the public and the public institution is needed [27, 28]. For these reasons, and in order to tackle the question we set out in this paper, we turn to political theory for accounts of public trust and specifically to the work of Mark Warren.

According to Warren, the term public trust denotes the trust warranted to public institutions tasked with providing a public good or service [29]. The basic conditions for public trust are convergent interests between the public and the institution, and commitment to serving and promoting the public good [29]. Democratic societies ought to be organised in a way that facilitates a clear separation between impartial bodies that provide public goods, and partial bodies, such as political branches of the government and political institutions (e.g. elected executives), which do not take on such commitments. According to Warren, partial bodies warrant public distrust based on the fact that their interests, motivations and intentions do not correspond with those of the general public, but only with certain sub-groups of the general population (e.g. party members) [29]. In other words, an institution’s *raison d’etre* to provide public good is what warrants public trust (even if it does not guarantee it). A commitment to public good can be seen as an indication of the good will such institutions hold towards the trustor, i.e. the public, and their moral motivation to validate the public’s dependency on them. On the other hand, public distrust towards partial institutions is based on the recognition that these institutions do not necessarily have good will towards the public or accept a commitment to provide a benefit for all (even if it could be argued that they might have good will towards certain subgroups that comprise the public, e.g. party members).

Warren maintains that a democratic society should welcome distrust towards partial institutions and put measures in place to facilitate and support it, namely, to institutionalise it [29]. This can happen by providing citizens the means with which to check and control the behaviour of partial bodies, to ensure that they do not harm public interest, and as much as possible align their activities with these that promote the public good. Institutionalising distrust can take the form of independent governance structures that provide checks and balances, monitor the actions of partial bodies, and introduce enforceable sanctions that they are meaningful enough to deter wrongdoing [29]. Furthermore, providing functional and appropriate forms of monitoring can prevent distrust from generalising and spilling over to impartial institutions [29]. One way of understanding the institutionalisation of distrust is as a guarantor of trust to the democratic system as a whole. It reinforces the idea that democracies are organised in such a way as to benefit the ‘demos’, rather than to benefit certain individuals or subgroups within society.

## The trust relationship between the public and the NHS

The NHS is widely regarded as a public institution that warrants and holds public trust. Its main function is to provide a public good, specifically to support and improve people’s health and wellbeing. From its inception in 1948 by Nye Bevan, the NHS’ key characteristic is its moral motivation as expressed in its solidaristic character [28, 30]. It is founded on a set of principles and values, which enjoy wide public support [31], that bind together the communities and people that it serves and the staff that work for it. As its Constitution states, ‘The NHS belongs to the people’ [32]. The NHS was founded on the principle that care should be delivered on the basis of need rather than on ability to pay. According to Veitch, this forms the basis of the relationship between the British public and the NHS; a public service user and public service provider with a shared understanding of the public good at stake [30]. In this relationship, the public has a moral and civic duty to fund the health service through taxation and use its resources prudently [32], while the State is obliged, through its appropriate financing and oversight of the healthcare service, to deliver appropriate care at the point of need. It is this stated commitment to serve the public good of healthcare that suggests an alignment of values and convergence of interests between the involved partners.

Although the latest British Social Attitudes survey demonstrates the public’s commitment and faith in the core principles of the NHS [31], public trust in its data sharing practices appears somewhat problematic. Various studies conducted across the years to gauge public attitudes to data sharing in healthcare and beyond have reported disquiet at the involvement of private companies in the healthcare space, with some voicing concerns about a potential “sell-off” of NHS patient data to private for-profit companies for research and product development [12–14, 33–40].

As it is often pointed out, the involvement of the private sector in the NHS is not a new phenomenon [41]. It can be traced back to the National Health Service and Community Care Act 1990, which separated the healthcare provision function of the NHS from the healthcare purchase function. Later, the Health and Social Care Act 2012 further fragmented the service, inviting more private companies into the healthcare space by introducing a tendering process for the commissioning of health care services under the principle of competition [30, 42]. More recently, the Health and Care Act 2022 represented a significant shift away from competition and towards collaboration between health providers [43, 44]. As some argue, whether these reforms will manage to halt the erosion of public services and the further involvement of private interests in the NHS depends on how and the context within which they will be implemented [43]. In any case, such changes can have a significant impact on the relationship between the public and the healthcare institution [30].

Similarly, since the introduction of IT medical systems and Electronic Health Records (EHR), questions about private involvement when it comes to health data access and sharing have increasingly come to the foreground. Existing surveys and research studies reveal snapshots of a complex picture about public attitudes towards data sharing; namely, while participants are generally open to the sharing of health data for the benefit of patients, the NHS and for the broader public benefit, there is consistent uneasiness associated with the sharing of health data with private organisations and the commercial exploitation of NHS data [12, 13, 33–39, 45]. As we demonstrate in the next section, the digitisation of the NHS and the broader datafication of healthcare along with the adopted ‘health and wealth’ agenda introduce new challenges in the context of healthcare, further impacting on the relationship between the public and the NHS.

## Digitisation and datafication of the NHS – the ‘health and wealth’ agenda

The digitisation of the NHS started in 2002 aiming (and missing) to achieve a ‘paperless’ NHS by 2018 and a ‘core level of digitisation’ by 2024 [46]. This process coincided with what Faulkner et al. call ‘the research turn’ in the NHS [47]. In 2003, the vision of UK’s Department of Trade and Industry’s Biotechnology Innovation and Growth Team [48], and later in 2006, the Department of Health’s publication *Best Research for Best Health* [8] made research a key element of the NHS, placing it at the centre of a national ‘health and wealth’ agenda. As the foreword of the latter report stated:

The vision that this strategy describes is underpinned by our determination to ensure that the NHS contribution to health research is a centrepiece of the Government’s ambition to raise the level of research and development (R&D) to 2.5% of GDP by 2014 (8).

The digitisation of the NHS and more specifically the introduction of Electronic Health Records (EHRs) were central to this strategic vision. Moving from paper records to highly mobile recorded data heralded an infrastructure and with it a new set of private partners that facilitated a ‘more entrepreneurial approach to data’ [47].

This entrepreneurial approach was further crystallised in 2012. The newly introduced pledge in the NHS Constitution in 2013 for England ‘to anonymise the information collected during the course of your treatment and use it to support research and improve care for others’ [49] was accompanied by the introduction of the Health and Social Care Act 2012 which established a new legal framework for the flow of patient information within the NHS [50]. While even before its introduction, there were special exemptions^1^ that facilitated (and continue to facilitate) the legal disclosure of patient-identifiable data to a third-party without consent, it was the 2012 Act that enabled the obligatory release-upon-request of patient information from every health and care provider in England directed by the then newly formed body, NHS England, which is an executive non-departmental public body sponsored by the Department of Health and Social Care [51, 52]. Following the intervention of the National Data Guardian, this was later accompanied by a (conditional) Opt Out option – which does *not* apply when data is needed for the purposes of individual care, and *may* only apply for any other purposes beyond individual care [53].^2^ Specifically, in relation to the use of health data beyond a patient’s individual care, the option to Opt Out only applies to identifiable data, and does not apply to anonymised data. As the NHS website states: Your choice [to opt out] does not apply, ‘[w]hen information that can identify you is removed’ [54].^3^ However, as it is becoming increasingly understood, removing identifiable information does not necessarily render data anonymous [55].^4^

These developments meant that information shared between doctors and patients in confidence was being transformed into usable streams of data that could be accessed by a range of actors for both primary and secondary uses, such as health services management and research, in an increasingly datafied landscape. Traditionally, when patients were enrolled in healthcare research, this would happen as part of specific and contained research projects, and with specific and individual consent. Similarly, anonymised data have long been offered for research and secondary purposes without the option of an Opt Out [57]. Both these practices continue to our day. However, the advent of the Big Data era with its major advancements in data science [58] and the subsequent datafication of healthcare [59, 60], along with the regulatory legal and policy changes, as briefly described above, have given rise to a more complex situation. As the next section demonstrates, the financialisation of healthcare complicates matters further [61, 62].

In 2013, the then UK Prime Minister David Cameron promised that everyone will be a research subject, by default [62]. Cameron’s promise was part of a broader vision - still persisting today - to facilitate research and innovation by making Britain a world leader in the life sciences sector and health-tech industry [6].^5^ The NHS with its longitudinal generational health data has become instrumental in the realisation of this vision, as consecutive UK governments have been strategising for ways not to ‘waste’ what is now seen as a valuable *asset* but exploit it as a way of competing in the global health knowledge market [63]. The assetisation of NHS data, through their transformation into a financial resource that can participate in a speculative economy, enables not only the financialisation of healthcare research and innovation [64] but also the further collapse of boundaries between research, innovation and the provision of healthcare. Nowadays, NHS patients, staff and the public as a whole are expected to support the NHS as the main healthcare providing institution, but also participate in ‘power[ing] the UK economy’ [65] by supporting the NHS’ role as ‘a major investor and wealth creator in the UK’ [7] as the boundaries between research and direct clinical care become increasingly blurred [61].

The aforementioned digitisation of the NHS and assetisation of NHS data coincide with the entry of new private commercial interests and companies in the healthcare space. Global consumer tech companies such as Google, Microsoft and Amazon, along with numerous other intermediaries and start-ups that were, up until very recently, alien to the healthcare space, can now buy, have access to or be handed over NHS patient data for the development of for-profit tools and products [66–68] and for the furthering of their own commercial strategies [69]. Furthermore, such private companies can secure strategic infrastructural positions acting as health ‘data intermediaries’ [69] or data ‘prospectors’ [70] as they provide the proprietary bricks and mortar of ‘essential infrastructures’ [71, 72]. As Prainsack notes, the increased digitisation of health and health-related activities allows these companies to perform a number of roles simultaneously, from producing devises used for patient monitoring to developing the software that collects and processes the data, which affords them not only a much greater role but also increasing power in this space [72, 73].

These developments have raised concerns about the ways in which healthcare data are utilised and the types of data sharing strategies employed resulting, in recent years, in some costly - both in monetary but also reputational terms - policy decisions [74, 14, 39, 45]. So, in 2016, and with more than a million of people opting out (when given the chance) [75], the care.data plan was abandoned. After its failure, some argued that what was needed was greater transparency and a better information campaign about the benefits of sharing/using NHS patient data [76]. However, as others have pointed out, attempts to address this perceived public trust deficit by trying to inform and educate people on the benefits of data sharing and new technologies fail to take into account the underlying reasons that lead to public distrust [3, 77]. So it is no surprise that when in May 2021 the government attempted to share NHS patient data with private companies for research and development under the General Practice Data for Planning and Research (GPDPR) scheme, concerns were raised once again. After more than a million people opted out of the scheme within a month of its announcement, again the government had to pause [10]. Although there are still plans for the GPDPR scheme to go ahead, it is an open question whether public trust will follow [14, 39, 45]. Recent concerns raised about the involvement of the controversial private company Palantir in key NHS data operations, during and beyond the pandemic, indicate that this issue is not going away any time soon [78, 79].

## Changing roles

Nye Bevan’s original vision for the NHS was of a healthcare system based on solidarity; namely, a principled relationship between the public, the health service and the State, within which certain rights but also duties and obligations for all parties involved emerge as a type of ‘communal responsibility of and for each and all’ for the provision of the public good that is healthcare [30]. This solidaristic character is still reflected in the NHS Constitution which outlines the rights and responsibilities of ‘how patients, the public and staff can help the NHS work effectively and ensure that finite resources are used fairly’ [49].

In recent decades, arguments have been made to defend a new way the public ought to discharge its solidarity-based obligation in the healthcare context and promote this public good, that is through (voluntary) participation in biomedical research, especially when participation is minimally risky and minimally invasive, such as submitting samples and data to a biobank [80–83]. Others, though, have rejected these claims suggesting that research participation can only be understood as an imperfect moral duty, rather than a strong moral obligation [84] raising justice-based concerns particularly in relation to who stands to benefit and whether research participation is the best way to promote the public good of healthcare or demonstrate solidarity [85–87]. Interestingly, even those defending research participation as a moral obligation do not go as far as to suggest that such an obligation should be mandatory or legally enforced, maintaining the importance of autonomy in this context [80, 82]. Yet, in the case of anonymised NHS data, the policy decision to make everyone a default research subject, in the sense that all patients’ anonymised data can potentially be shared, bypasses these ethical debates and forecloses the normative conclusion. In the monopolistic healthcare system that is the NHS, participation in the particular version of research and innovation, and in the broader ‘health and wealth’ agenda, becomes not just a moral duty, but an unavoidable civic obligation inextricably tied with the public’s ability to access healthcare, and part of the solidarity-based relationship between the public and the healthcare service.

The adoption and operationalisation of the ‘health and wealth’ agenda in England stealthily, yet fundamentally, changes the role of the NHS; from a public institution tasked with the provision of healthcare to one tasked with (also) using its position as the main healthcare provider to generate a resource to promote research and innovation, including in the private sector. This change inevitably impacts on the relationship between the public and the NHS. Nowadays, members of the public are not just citizens of a welfare state who support the system through taxation and prudent use of resources, but also data subjects enlisted in supporting a particular approach to research and innovation merely by virtue of seeking healthcare [63]. In this sense, the public is required to “pay twice” for their healthcare, once through taxation but also through their data. In this new relationship, patients are both citizens of the welfare state (whose taxes are still funding the healthcare service) and also datafied entities whose data are the asset to be traded in this new economy, as the roles of citizen, patient and data subject merge together.

And it is not just the public that seem to have acquired a multiplicity of roles in this new healthcare landscape. The healthcare service itself is also taking on new aims and objectives, including stimulating economic growth and supporting private sector collaborations [88]. The expansion of the role the NHS plays as a public institution in society is neither straightforward nor uncontroversial. The fact that different governments even within the UK have chosen to adopt different strategies when it comes to managing health data demonstrates that co-opting a solidarity-based public healthcare system to support a wealth-generating agenda is a political choice rather than a socio-economic inevitability [89]. This change unites the pursuit of universal healthcare as a public good with the aims of successive UK governments to derive wealth based on a particular neoliberal model of bioeconomy [43, 90]. As such, and to return to Warren’s analysis of public trust, it mixes an impartial public and trusted institution with a partial one that seeks to secure its political goals using the former as its means [29]. Furthermore, it brings into question the NHS’ commitment to serving and promoting the public good of healthcare, as it forces it to endorse multiple aims, including the generation of wealth for the private sector. The cost, as cases such as the implementation of care.data demonstrate, is the growth of distrust resulting in the seemingly contradictory situation where the public declares its trust for the NHS to handle its data [31], while, given the opportunity, it votes with its feet when such initiatives are introduced.

## Next steps

So far, we have seen that the public is committed to healthcare as a public good and continues to support a national healthcare service that is dedicated at providing care to all according to need [31]. They are happy to support this service both with their taxes as well as with their data, as long as it is for the “greater public good” [12, 91]. At the same time, they are repeatedly objecting to a version of the NHS that uses patient data to promote economic and industrial growth including in the private sector. For example, a systematic review of public opinions on the use of patient data for research in the UK and Republic of Ireland showed that the public’s support is widespread yet conditional upon competence in keeping data secure, and free from interference of “private interests” [91]. In their workshops on patients’ views of the possible benefits from reuse of personal health data, Aitken et al. note that none of their participants ‘spoke of societal benefits in terms of economic benefit’ [92]. Furthermore, earlier work has shown that regardless of their success in attaining targets and improving cost-efficiency, NHS reform programmes that threaten the legitimacy of the public service are met with public distrust and unease [93].

Following our analysis, the problem that emerges is one of misalignment of aims and values between the public and the healthcare services that impacts on the trust relationship. By adding further economic aims into the function of the NHS, the commitment to health as the main and sole public good served by this public institution is questioned. Under the ‘health and wealth’ agenda, the health service is required to promote multiple goals, and it is this that introduces distrust into the relationship. Following Warren’s theory that partial institutions do not warrant public trust suggests that it is not possible for the NHS to behave like a partial institution, one that serves aims beyond that of promoting the public good, and still warrant public trust [29].

Healthcare is widely accepted as a public good and public healthcare institutions, such as the NHS, are founded on that understanding. The ‘health and wealth’ policy agenda adopted by successive governments in the past couple of decades disrupts this understanding as it collapses one term onto the other. The datafication of healthcare, as it is currently pursued in England, further reinforces this relationship as illustrated by the recent *Data Saves Lives* policy report which proclaims: ‘So that we can continue to provide the best care for the citizens we serve, we must safely grasp the opportunities for data-driven innovation […] and power the UK economy’ [65].

However, recent reports in the complex and fragmented landscape of NHS data reveal that the ways in which NHS data are shared with external and private actors, and the extent to which any benefits that derive from this data then return to the NHS and the general public, if at all, is far from straightforward [67, 68]. This demonstrates that, despite its rhetorical neatness, the formulation data = wealth = health that many policy reports, such as the aforementioned *Data Saves Lives* [65], appear to assume needs to be carefully examined and robustly demonstrated, rather than just wishfully proclaimed, especially if it is to convince a sceptical public. As such an important theoretical question that needs to be investigated is the following; under what conditions could the solidarity-based obligation to promote health between the public and the State be reasonably expected to also include the promotion of research and innovation? This is a question which cannot be settled merely by arguing that the public has a moral obligation to participate in research. It needs to be demonstrated that this is not just an imperfect or weak obligation, but one that should become a mandatory civic duty, part of the solidarity-based relationship between the national health institution and the public. This would require both philosophical and empirical work. For example, in order for such an expansion of aims to be acceptable within the existing relationship between the public and the health service, one should examine whether these multiple goals are compatible with each other or whether they might lead to the corruption or corrosion of existing and accepted values and priorities [94]. What are the consequences of taking on multiple aims for the provision of care on the ground and how should conflicts between achieving these different aims be resolved?

Furthermore, one would need to explore whether and to what extent these activities, as they are currently pursued, serve, directly and primarily, the public good of healthcare. Are these activities the most effective and efficient ways to promote health, as opposed to other types of social interventions? Is research and innovation, as currently practiced, able to address existing and widespread social, economic and political inequalities, thus making promoting research and innovation the preferred expression of civic or even global solidarity? The latter is particular pertinent as there are many who argue that tackling inequalities in the distribution of power, money and resources, and improving the conditions in which people are born, grow, live, work and age can do more to promote the health of the public rather than investment in research and in data-intensive health technologies like genomics and AI [85, 86, 95].

Finally, and once we have a clearer theoretical and empirical understanding of these issues, it would be necessary for an honest and informed public debate to take place to ascertain whether this expansion of the aims served by the NHS should become part of a new social contact. More fundamentally, it needs to be determined through public dialogue, what values should be endorsed and promoted by a public institution like the NHS. Would the public be ready and willing to support the changing character of the NHS and adopt its new role in this relationship? And, most importantly, would it still be able to trust it?

## Conclusion

This paper set out to address the question, what is it about sharing NHS data for research and innovation that challenges public trust? In order to do so, it drew from political theory to provide an account of public trust that helps better understand the relationship between the public and the NHS within a democratic context, as well as, the kind of obligations and expectations that govern this relationship. After examining whether the ways in which the NHS is managing patient data along with its collaboration with the private sector fit under this trust-based relationship, it argued that the digitisation of the NHS and the broader ‘health and wealth’ agenda adopted by consecutive UK governments represent a major shift in the institutional character of the NHS. We demonstrate that this shift brings into question the meaning of public good the NHS is expected to provide, hence challenging public trust. In conclusion, the paper argues that in order to address the problem of public trust the following are needed: (a) a theoretical and empirical examination of the benefits but also the costs associated with this shift, (b) an open conversation at a public level to determine what values a public institution like the NHS should promote.

## Data Availability

This manuscript is based on the review and analysis of relevant literature. It does not contain any original data.

## Footnotes

1 As Cheung explains, ‘Access to [identifiable] health data without consent is possible in England and Wales through Sect. 251 of the NHS Act 2006 and its current regulations (usually referred to as ‘Sect. 251’). Through this process, the Common Law of Confidentiality is temporarily set aside for the specific purpose applied for, although responsibilities resulting from the Data Protection Act are still applicable (e.g. the obligation to be ‘lawful, fair and transparent’).’ [28].

2 While the option of an Opt-Out was nominally offered, Vezyridis gives an account of the problems, complications and emissions that rendered this option problematic when the care.data scheme was introduced. These include lack of public information on its availability and the inability to process the high number of subsequent Opt Outs. Although a number of steps were taken in an attempt to address these issues, the care.data system was finally withdrawn [28].

3 The National Data Opt-out Operational Policy Guidance offers a more comprehensive explanation: ‘The opt-out does not apply when the individual has consented to the sharing of their data or where the data is anonymised in line with the Information Commissioner’s Office (ICO) Code of Practice on Anonymisation.’ [53].

4 Also see Meszaros for a detailed account of how the data protection terminology in key policy documents and guidelines in the UK contains inconsistencies leading to confusion between the terms anonymization/de-identification/pseudonymisation, challenging the soundness of this regulatory framework [56].

5 As the Health and Social Care Act 2012 has been superseded by the Health and Social Care Act 2022, the political appetite to facilitate research and innovation through the use of NHS data remains. The new Act merges the NHSX and NHS Digital into NHSE/I. This move has been described as ‘a significant retrograde step in defending the rights of citizens with respect to the collection and use of their health data. And has the potential for undermining the relationship between clinicians and their patients’ [63].
