# Correction to: Data-driven research and healthcare: public trust, data governance and the NHS

**DOI:** 10.1186/s12910-023-00955-4

**Published:** 2023-10-04

**Authors:** Angeliki Kerasidou, Charalampia (Xaroula) Kerasidou

**Affiliations:** 1 Ethox Centre, Oxford Population Health (Nuffield Department of Population Health, Big Data Institute, Li Ka Shing Centre for Health Information and Discovery Oxford, University of Oxford, Oxford, UK; 2 Ethox Centre, Oxford Population Health (Nuffield Department of Population Health, Big Data Institute, Li Ka Shing Centre for Health Information and Discovery Oxford, University of Oxford, Oxford, UK

Following publication of the original article [1], in this article some references were wrongly updated and now it has been corrected.

The original article has been corrected.

## References

[1] Kerasidou A, Kerasidou C. Data-driven research and healthcare: public trust, data governance and the NHS. BMC Med Ethics 2023;24:51. 10.1186/s12910-023-00922-z 10.1186/s12910-023-00922-z PMC10349411 37452393

